# Preventive effect of insect tea primary leaf (*Malus sieboldii* (Regal) Rehd.) extract on D‐galactose‐induced oxidative damage in mice

**DOI:** 10.1002/fsn3.1821

**Published:** 2020-08-13

**Authors:** Ruokun Yi, Xi Chen, Wenfeng Li, Jianfei Mu, Fang Tan, Xin Zhao

**Affiliations:** ^1^ Chongqing Collaborative Innovation Center for Functional Food Chongqing University of Education Chongqing China; ^2^ Chongqing Engineering Research Center of Functional Food Chongqing University of Education Chongqing China; ^3^ Chongqing Engineering Laboratory for Research and Development of Functional Food Chongqing University of Education Chongqing China; ^4^ Intensive Care Unit The First People's Hospital of Chongqing Liang Jiang New Area Chongqing China; ^5^ School of Life Science and Biotechnology Yangtze Normal University Chongqing China; ^6^ Department of Public Health Our Lady of Fatima University Valenzuela Philippines

**Keywords:** D‐galactose, insect tea, mRNA, oxidative damage

## Abstract

Insect tea is consumed as a health beverage in China. The insect tea primary leaf (ITPL) is rich in bioactive substances, which are also used as traditional Chinese medicine. This study investigated the role of ITPL in reducing the oxidative response induced by D‐galactose in mice. Mice were intraperitoneally injected with D‐galactose to induce oxidative damage. The effect of ITPL was tested by pathological observation, serum detection with kits, quantitative polymerase chain reaction, and Western blot. The experimental results show that ITPL increased the thymus, brain, heart, liver, spleen, and kidney indices of oxidized mice. ITPL increased superoxide dismutase, glutathione peroxidase, and glutathione levels and reduced nitric oxide and malondialdehyde levels in the serum, liver, and spleen in oxidative damaged mice. The pathological observations show that ITPL reduced the oxidative damage of the liver and spleen in mice induced with D‐galactose. Simultaneously, ITPL upregulated mRNA expression of neuronal nitric oxide synthase, endothelial nitric oxide synthase, cuprozinc‐superoxide dismutase, manganese superoxide dismutase, catalase, heme oxygenase‐1, nuclear factor‐erythroid 2 related factor 2, γ‐glutamylcysteine synthetase, and NAD(P)H dehydrogenase [quinone] 1, and downregulated the expression of inducible nitric oxide synthase in the liver and spleen of oxidized mice. ITPL had beneficial preventive effects on the oxidative damage caused by D‐galactose in mice and was more effective as an antioxidant than vitamin C. The component analysis test by high‐performance liquid chromatography indicated that ITPL contained the following seven compounds: neochlorogenic acid, cryptochlorogenic acid, rutin, kaempferin, isochlorogenic acid B, isochlorogenic acid A, and hesperidin. ITPL is a plant with excellent antioxidant activities derived from its bioactive substances.

## Graphical abstract

Insect tea primary leaf (*Malus sieboldii* (Regal) Rehd.) extract could inhibit D‐galactose‐induced oxidative damages in mice.

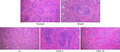

## 1 INTRODUCTION

Insect tea is a traditional health beverage in southern China. The process of producing insect tea is specific. Insect larvae ingest leaves and produce excrement, and the larval excrement is processed to produce insect tea (Zhao et al., 2018). In addition to being affected by the insect species, the quality of insect tea is determined by the type of food ingested by the larvae. The plants used to produce insect tea include Kuding tea (*Ligustrum lucidum*), Liubao tea (*Camellia sinensis*), and Holly (Liu, Yang, Shang, Hu, & Wang, 2013). *Malus sieboldii* (Regal) Rehd is a raw leaf used to produce insect tea. The fruits and leaves of *M. sieboldii* are used as Chinese medicine to aid digestion and reduce inflammation (Wang, Meng, Mei, & Xiao, 2006). An abundance of active ingredients is found in *M. sieboldii* (Regal) Rehd leaves, including amino acids, polysaccharides, flavonoids, and saponins (Wen, Shen, Zang, & Hu, 2004; Zeng, Yang, & Yang, 2019).

Oxidative stress is a normal phenomenon in the body. It is a gradual process from the time of birth, which can cause sustained harm to individuals. Oxidative stress in the body induces and exacerbates many diseases, such as hypertension, Parkinson's disease, and atherosclerosis (Buford, 2016; Chard et al., 2017; Kitada, Ogura, & Koya, 2016). The abnormal accumulation of active free radicals in vivo causes oxidative damage to the biomacromolecules that comprise the tissues, leading to oxidative stress. The degree of oxidative damage in tissues increases as oxidative stress is increase (Rao, 2009). Regulating the level of oxidative stress in the body is important to maintain normal functioning and overall health (Hohensinner et al., 2018). Consuming a small amount of D‐galactose will not damage the body, as it is converted to glucose, but consuming a large amount of D‐galactose affects normal metabolism and produces a large amount of superoxide anions and oxidation products, which gradually cause oxidative damage to the body. Therefore, D‐galactose is often used to experimentally induce oxidative damage (Li, Xia, Yang, & Zhong, 2013; Li et al., 2016). The oxidation model established by D‐galactose to verify the antioxidative activity of antioxidant active substances has been applied to develop health products.

Many plants have antioxidant effects, such as plants that produce tea (Carluccio et al., 2003; Sharma et al., 2016). These plants contain primarily three antioxidant enzymes, such as superoxide dismutase (SOD), glutathione peroxidase (GSH‐Px), and catalase (CAT) as well as polyphenols that act as antioxidants (Delwing‐Dal Magro et al., 2016).

After extracting the active substances from the primary leaves of chrysanthemum tea and applying it to D‐galactose to induce oxidation in mice, we observed the effects of insect tea primary leaf (ITPL) on oxidized murine serum and tissue samples. Subsequently, we analyzed and clarified the mechanism by which ITPL acts to prevent oxidation. We accumulated information on the theoretical basis to perform additional research on the human body and for industrial development.

## 2 MATERIALS AND METHODS

### 2.1 ITPL extraction

The original insect tea leaves were freeze‐dried (Changsha Sanye Insect Tea Co., Ltd.); 500 g of ITPL was crushed, 5 L of distilled water was added, the ITPL was extracted at 80°C for 2 hr, collected, and filtered. An additional 5 L of distilled water was added to the final product residue, and it was re‐extracted at 80°C for 2 hr. The two filtrates were combined, the methanol was removed with a rotary evaporator, and the extracts were collected (Qian et al., 2018).

### 2.2 Experimental design

**FIGURE 1:**
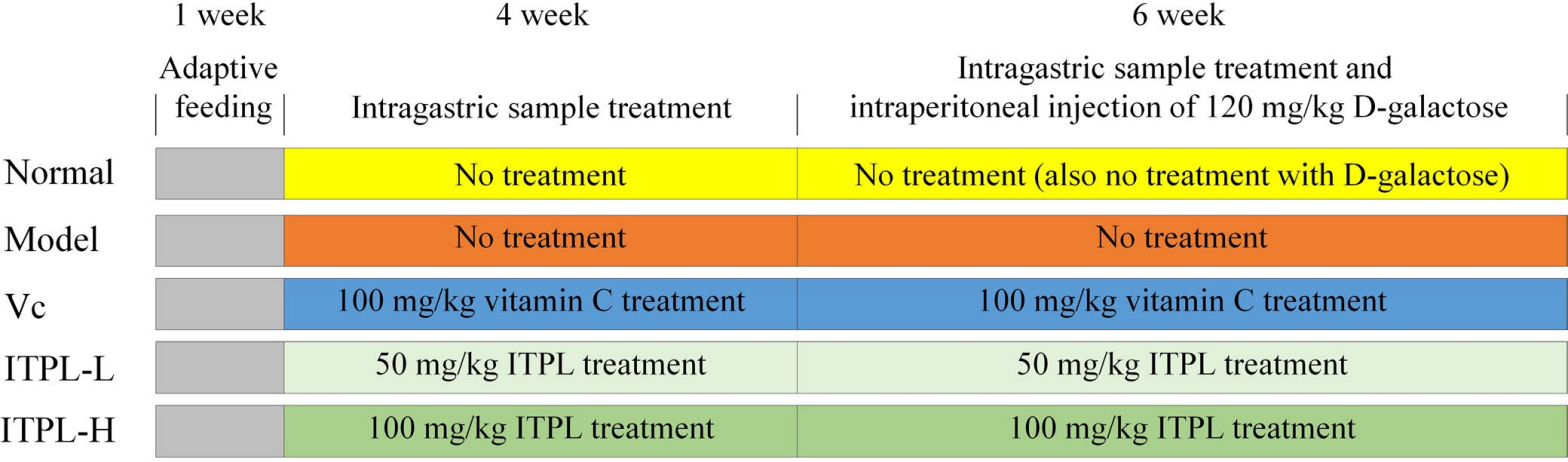
Animal experimental processes in this study

A total of 25 male and 25 female 6‐week‐old ICR mice were purchased from Chongqing Tengxinbil Experimental Animal Sales Co., Ltd. The mice were divided into five groups. The experiment was conducted for 10 weeks (Figure 1), after which all of the mice were killed following a 24‐hr fast (Qian et al., 2018). Serum samples were obtained from the heart, and samples of the internal organs were excised for the experiment. The organ indices of the thymus, brain, heart, liver, spleen, and kidney were measured as follows: organ index = organ mass (g)/mouse body mass (kg) × 100. The protocol for these experiments was approved by the Ethics Committee of Chongqing Collaborative Innovation Center for Functional Food (201904022B), Chongqing, China. In this study, the welfare of laboratory animals was treated in accordance with the European Union 2010/63/EU directive on the protection of laboratory animals.

### 2.3 Measurement of nitric oxide (NO) and malondialdehyde (MDA) levels and the activities of SOD and GSH‐Px in serum and liver tissue samples

The extracted murine plasma was centrifuged at 2504 *g* for 10 min, and the serum in the upper layer was separated and extracted. The murine serum contents were measured according to the manufacturer's recommendations (Shanghai Jingkang Bioengineering Co., Ltd.). The murine livers consisting of 10% homogenates were centrifuged at 2143 *g* for 15 min. The supernatant was obtained to measure liver tissue contents according to the reagent method.

### 2.4 Histological observations of the liver and spleen tissues

Murine liver and spleen tissues (0.5 cm^2^) were extracted and fixed in 10% formalin solution for 48 hr. The liver and spleen tissues were dehydrated, rendered optically transparent, immersed in wax, embedded, and sectioned for hematoxylin and eosin staining. We observed the changes in tissue morphology using an optical microscope (XSP‐BM17, Shanghai Telun Optical Instrument Co., Ltd.) (Zhou et al., 2019).

### 2.5 Quantitative polymerase chain reaction (PCR) assay

Total RNA of liver and spleen tissues from the mice was extracted using RNAzol (Thermo Fisher Scientific, Inc.). The concentration of the extracted total RNA was diluted to 1 μg/μl, and 5 μl of the diluted total RNA solution was used in the assay. To obtain a cDNA template, extraction and reverse transcription were performed according to the manufacturer's instructions. The following procedure was performed for 40 cycles: 2 μl of cDNA template and 10 μl of SYBR Green PCR Master Mix, 1 μl of upstream and downstream primers (Thermo Fisher Scientific) were mixed and reacted for 60 s at 95°C, and 15 s at 95°C, 30 s at 55°C, and 33 s at 72°C, respectively. Finally, they were both tested for 30 s at 95°C and 35 s at 55°C, internally referred to GAPDH. The 2^−ΔΔCt^ method was used to calculate expression (Liu, Zhang, et al., 2019).

### 2.6 High‐performance liquid chromatography

We dissolved ITPL in dimethyl sulfoxide to obtain a 10 mg/ml solution, which we diluted with 50% methanol to obtain a 2 mg/ml ITPL solution sample. We filtered the ITPL sample and the standard solution through a 0.22 μm membrane and assayed a sample volume of 10 μl. The chromatographic conditions included a Hypersep C18 column (4.6 mm × 150 mm, 5 μm); mobile phase A, 0.5% acetic acid water; mobile phase B, acetonitrile; flow rate, 0.5 ml/min; column temperature, 25°C; and detection wavelength, 280 nm (Ultimate 3000, Thermo Fisher Scientific) (Li et al., 2019).

### 2.7 Statistical analysis

The trials were conducted three times for each test object. We analyzed the average values using SAS 9.1 statistical software (SAS Institute). One‐way analysis of variance was performed to determine whether the differences between the groups were significantly different at *p* < .05.

## 3 RESULTS

### 3.1 Organ indices

Table 1 shows that the thymus, brain, heart, liver, spleen, and kidney indices were highest in the normal group, while those of the experimental group were the lowest. Both ITPL and vitamin C significantly (*p* < .05) increased the organ indices of mice in the model group with oxidative damage. Additionally, the effects of ITPL were significantly superior to those of vitamin C at the same concentration. In general, ITPL inhibited the decrease in the organ indices caused by oxidative damage.

**TABLE 1:** Visceral tissue index of the experimental mice

Group	Thymus index	Brain index	Cardiac index	Liver index	Spleen index	Kidney index
Normal	0.22 ± 0.02^a^	2.06 ± 0.03^a^	5.18 ± 0.06^a^	41.05 ± 0.34^a^	4.32 ± 0.21^a^	14.19 ± 0.17^a^
Model	0.10 ± 0.01^e^	1.15 ± 0.02^e^	4.01 ± 0.04^e^	27.02 ± 0.26^e^	2.11 ± 0.26^e^	8.33 ± 0.15^e^
Vc	0.16 ± 0.02^c^	1.61 ± 0.04^c^	4.69 ± 0.08^c^	34.12 ± 0.30^c^	3.35 ± 0.22^c^	11.04 ± 0.13^c^
ITPL‐L	0.14 ± 0.02^d^	1.38 ± 0.03^d^	4.27 ± 0.05^d^	31.12 ± 0.22^d^	2.79 ± 0.14^d^	10.25 ± 0.11^d^
ITPL‐H	0.19 ± 0.01^b^	1.88 ± 0.02^b^	4.88 ± 0.05^b^	36.23 ± 0.24^b^	3.89 ± 0.17^b^	13.17 ± 0.20^b^

Note

±Standard deviation. ^a–d^Different superscripts indicate significant differences (*p* < .05) according to Duncan's multiple range test. Vc: gavage 100 mg/kg vitamin C; ITPL‐L: gavage 50 mg/kg insect tea primary leaf; ITPL‐H: gavage 100 mg/kg insect tea primary leaf.

### 3.2 Levels of NO, SOD, GSH‐Px, GSH, and MDA

GSH possessed the most powerful activity among the SOD, GSH‐Px, and GSH activities in the serum, liver, and spleen of the mice from the normal group (Tables 2, 3, 4), but the lowest levels of NO and MDA. In the experimental group, SOD, GSH‐Px, and GSH demonstrated the weakest activities with the highest NO and MDA levels. After ITPL, SOD, GSH‐Px, and GSH increased (*p* < .05) in the oxidative injured mice, whereas NO and MDA levels decreased significantly (*p* < .05). After ITPL‐H, the NO, SOD, GSH‐Px, GSH, and MDA levels of the experimental mice approached those of the normal group, revealing significantly better effects than those of vitamin C.

**TABLE 2:** Mouse serum biological indices

Group	NO (μmol/L)	SOD (U/ml)	GSH‐Px (U/ml)	GSH (mg/L)	MDA (nmol/ml)
Normal	14.89 ± 0.31^e^	259.68 ± 13.29 ^a^	217.88 ± 14.52^a^	48.12 ± 0.66^a^	2.79 ± 0.22^e^
Model	59.22 ± 1.17^a^	54.18 ± 4.83^e^	74.05 ± 5.01^e^	7.52 ± 0.48^e^	31.28 ± 0.77^a^
Vc	30.67 ± 1.25^c^	157.82 ± 10.12^c^	144.19 ± 9.36^c^	30.82 ± 0.59^c^	10.56 ± 0.50^c^
ITPL‐L	42.18 ± 1.06^b^	92.08 ± 8.80^d^	99.15 ± 7.80^d^	19.25 ± 0.63^d^	16.75 ± 0.49^b^
ITPL‐H	20.55 ± 0.71^d^	205.63 ± 12.17^b^	185.91 ± 10.85^b^	39.71 ± 0.56^b^	6.12 ± 0.40^d^

Note

±Standard deviation. ^a–e^Different superscripts indicate significant differences (*p* < .05) according to Duncan's multiple range test. Vc: gavage 100 mg/kg vitamin C; ITPL‐L: gavage 50 mg/kg insect tea primary leaf; ITPL‐H: gavage 100 mg/kg insect tea primary leaf. The following figure and table are the same.

**TABLE 3:** Mouse liver tissue indices

Group	NO (μmol/gprot)	SOD (U/mgprot)	GSH‐Px (U/mgprot)	GSH (mg/ gprot)	MDA (nmol/mgprot)
Normal	2.10 ± 0.12^e^	98.23 ± 5.83^a^	174.53 ± 10.23^a^	12.05 ± 0.69^a^	1.25 ± 0.11^e^
Model	9.87 ± 0.45^a^	20.85 ± 3.82^e^	62.08 ± 4.56^e^	1.82 ± 0.41^e^	9.63 ± 0.41^a^
Vc	4.26 ± 0.26^c^	71.25 ± 5.02^c^	121.05 ± 7.86^c^	6.22 ± 0.39^c^	4.08 ± 0.30^c^
ITPL‐L	7.11 ± 0.39^b^	37.89 ± 5.21^d^	91.02 ± 5.88^d^	3.28 ± 0.43^d^	7.82 ± 0.26^b^
ITPL‐H	3.15 ± 0.20^d^	83.26 ± 5.11^b^	150.58 ± 9.12^b^	8.91 ± 0.36^b^	1.87 ± 0.12^d^

Note

±Standard deviation. ^a–e^Different superscripts indicate significant differences in data (*p* < .05) according to Duncan's multiple range test. Vc: gavage 100 mg/kg vitamin C; ITPL‐L: gavage 50 mg/kg insect tea primary leaf; ITPL‐H: gavage 100 mg/kg insect tea primary leaf. The following figure and table are the same.

**TABLE 4:** Mouse spleen tissue indices

Group	NO (μmol/gprot)	SOD (U/mgprot)	GSH‐Px (U/mgprot)	GSH (mg/ gprot)	MDA (nmol/mgprot)
Normal	1.48 ± 0.09^e^	88.47 ± 7.10^a^	139.24 ± 12.53^a^	8.97 ± 0.62^a^	0.66 ± 0.10^e^
Model	9.10 ± 0.41^a^	16.59 ± 4.89^e^	35.78 ± 2.97^e^	1.66 ± 0.42^e^	6.97 ± 0.32^a^
Vc	4.56 ± 0.29^c^	55.45 ± 6.17^c^	88.12 ± 7.89^c^	4.97 ± 0.51^c^	2.50 ± 0.16^c^
ITPL‐L	6.58 ± 0.39^b^	28.79 ± 4.50^d^	50.36 ± 5.98^d^	3.12 ± 0.45^d^	4.43 ± 0.43^b^
ITPL‐H	2.11 ± 0.28^d^	69.11 ± 4.43^b^	111.05 ± 7.42^b^	6.35 ± 0.47^b^	1.21 ± 0.19^d^

Note

±Standard deviation. ^a–e^Different superscripts indicate significant differences (*p* < .05) according to Duncan's multiple range test. Vc: gavage 100 mg/kg vitamin C; ITPL‐L: gavage 50 mg/kg insect tea primary leaf; ITPL‐H: gavage 100 mg/kg insect tea primary leaf. The following figure and table are the same.

### 3.3 Histological observations of the liver and spleen tissues

**FIGURE 2:**
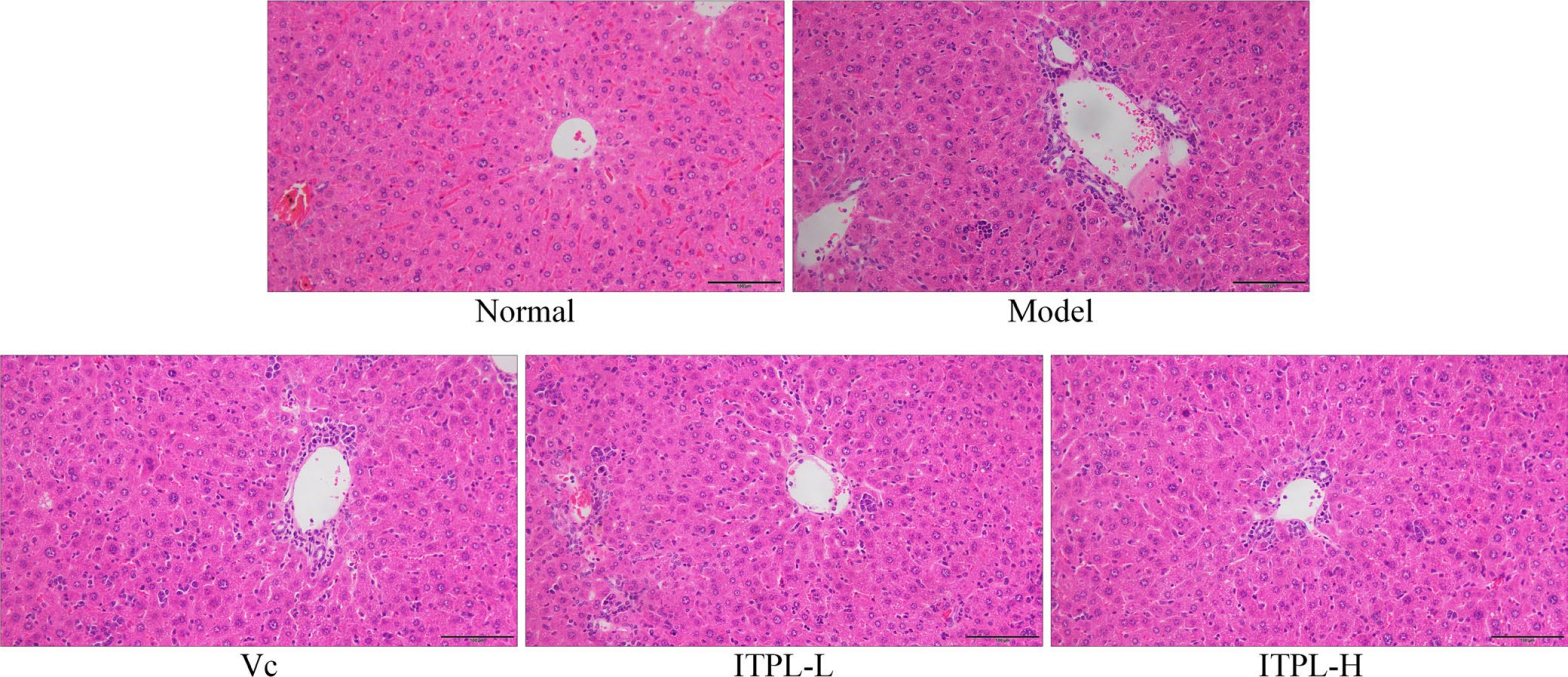
Observations of mouse liver stained sections (100×)

Figure 2 shows that the hepatocytes in the normal group were arranged radially around the central vein, with a regular and orderly morphology. The hepatocytes were the same size, with no evidence of aggregated inflammatory cells. The liver cells were disordered in the model group, their morphology was irregular, the boundaries between them appeared blurred, they were swollen, and several cells had incomplete nuclei. Inflammatory infiltration was evident in a large number of cells. ITPL effectively ordered the hepatocytes in the oxidized mice, arranged the liver cord relatively neatly, protected the hepatocytes, and clarified the cell structure. ITPL‐H transformed the morphology of the liver cells of the oxidized mice into a form more similar to the normal group.

**FIGURE 3:**
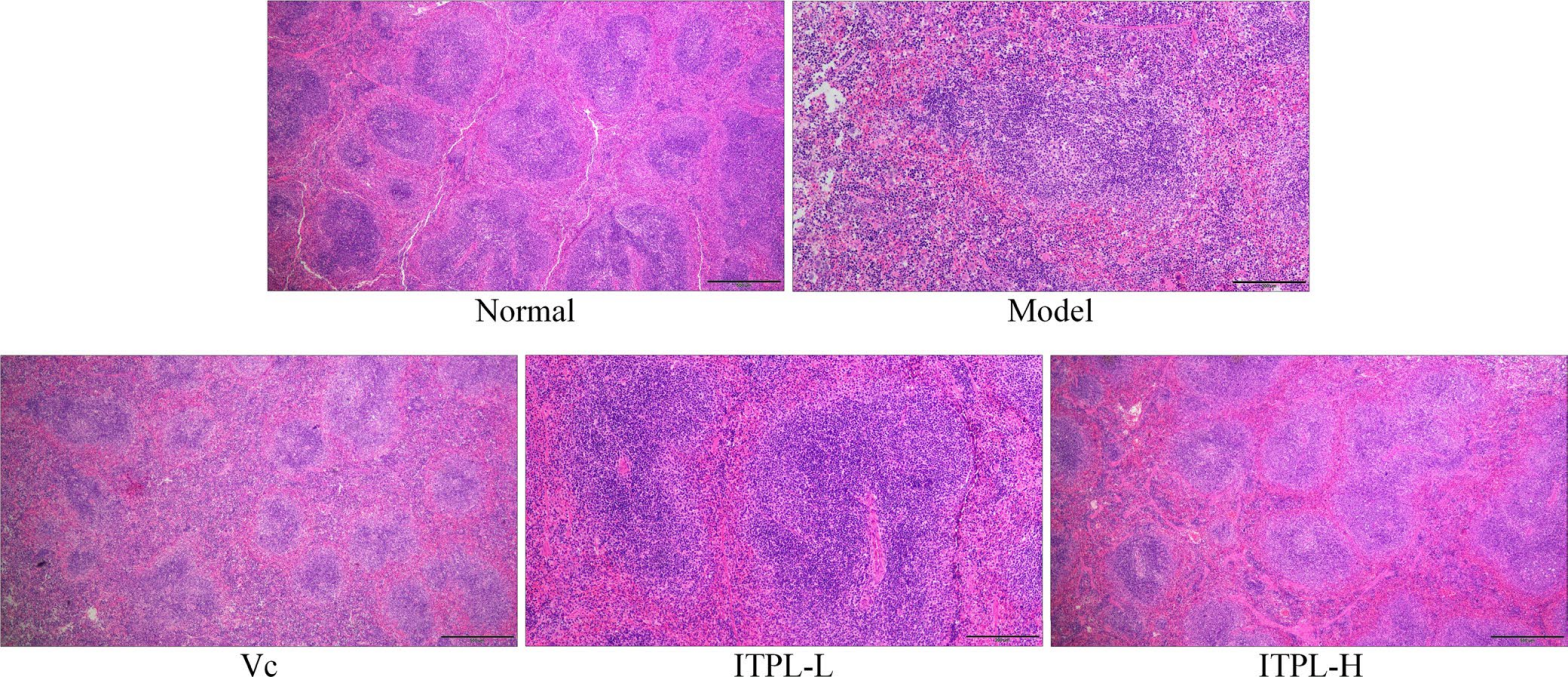
Observations of mouse spleen stained sections (100×)

Figure 3 shows that the structure of the murine spleen tissues from the normal group was orderly and complete. The skin*–*medullary junctions were clear, and the cells were arranged neatly. In the normal group, the structure of the spleen was comprised of irregularly shaped cells. The red pulp sinuses were dilated and filled with red blood cells. ITPL effectively alleviated these changes in the spleen tissues caused by D‐galactose. ITPL inhibited the lesions of the skin, liver, and spleen, caused by oxidation, and the effects were better than those of vitamin C.

### 3.4 Expression of nNOS, eNOS, and iNOS mRNA in liver and spleen tissues

**FIGURE 4:**
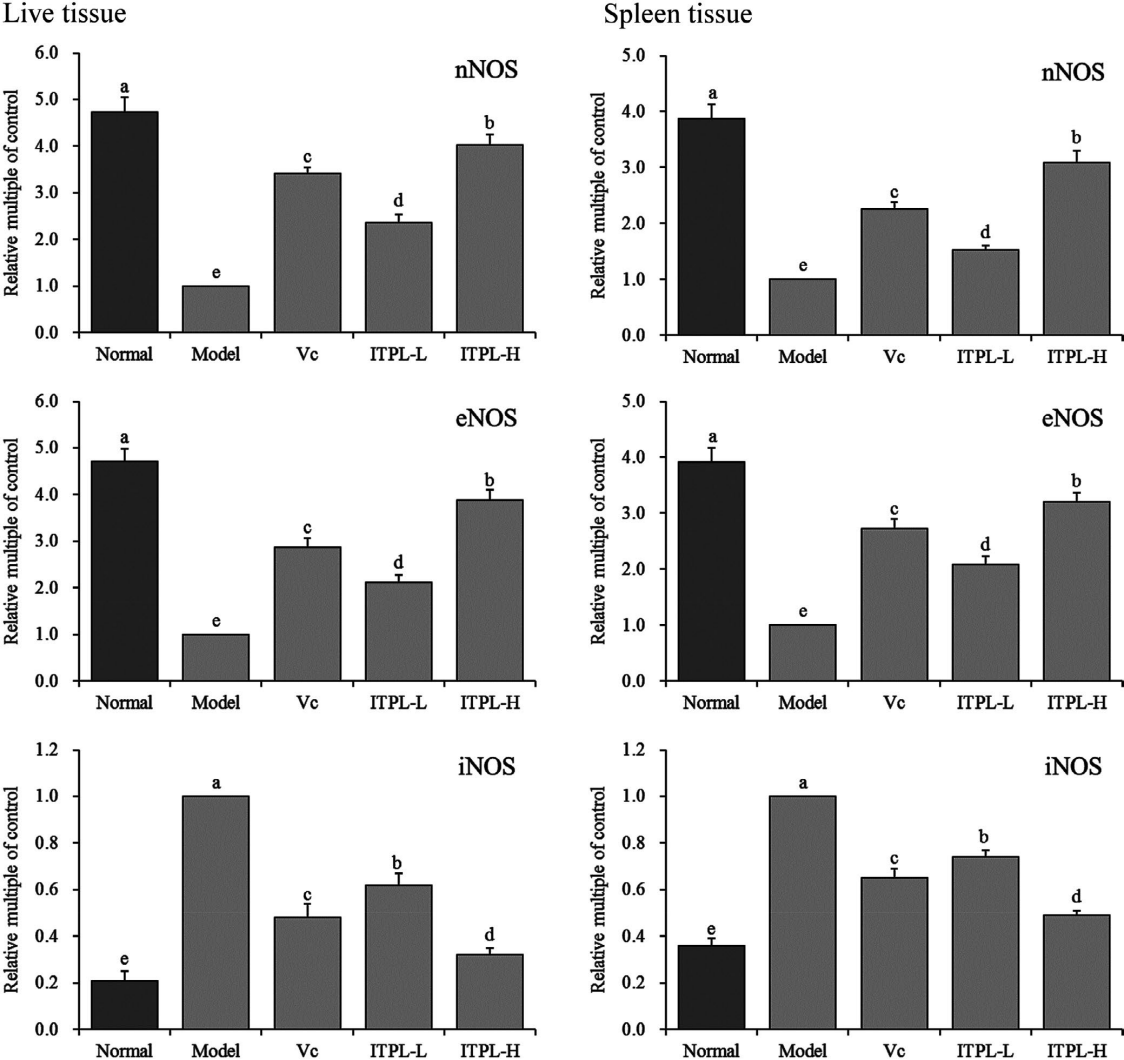
The nNOS, eNOS, and iNOS mRNA expression in liver and spleen tissues of mice. ^a–e^Different superscripts indicate significant differences (*p* < .05) according to Duncan's multiple range test. Vc: gavage 100 mg/kg vitamin C; ITPL‐L: gavage 50 mg/kg insect tea primary leaf; ITPL‐H: gavage 100 mg/kg insect tea primary leaf

Figure 4 shows that the expression levels of nNOS and eNOS mRNA in the liver and spleen tissues of mice in the normal group were significantly (*p* < .05) higher than those in the other groups, while the expression level of iNOS was significantly (*p* < .05) lower than that in the other groups. D‐galactose‐induced oxidation significantly reduced the expression levels of nNOS and eNOS mRNA in the liver tissues of the model group, but considerably increased the expression of iNOS. ITPL significantly (*p* < .05) inhibited the D‐galactose‐induced changes in the mRNA expression of liver tissues, and mRNA expression by the liver tissues of the oxidized mice normalized. The effects were better than those of vitamin C under the same concentration.

### 3.5 Expression of Cu/Zn‐SOD, Mn‐SOD, and CAT mRNA in the liver and spleen tissues

**FIGURE 5:**
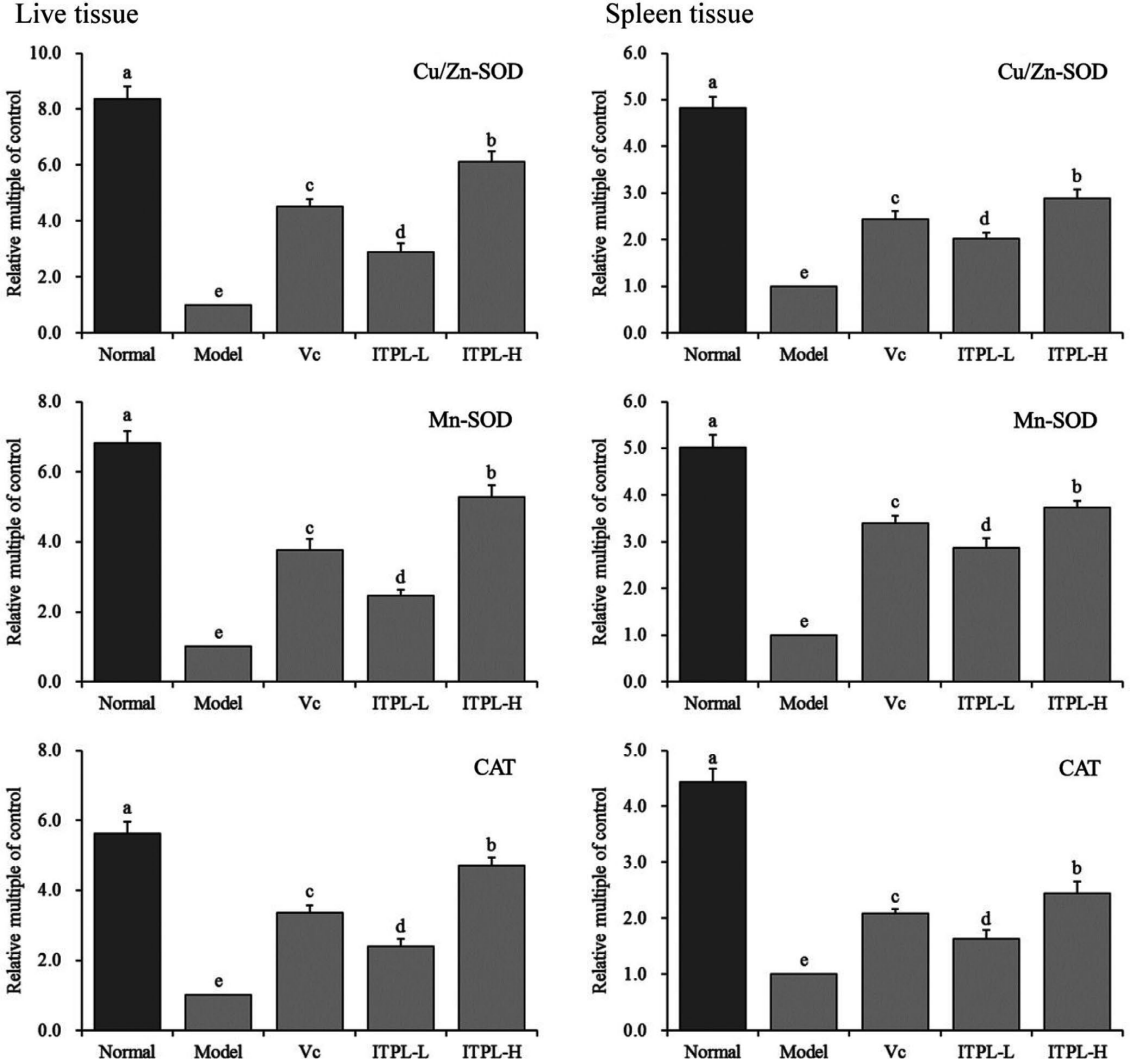
The Cu/Zn‐SOD, Mn‐SOD, and CAT mRNA expression in liver and spleen tissues of mice. ^a–e^Different superscripts indicate significant differences (*p* < .05) according to Duncan's multiple range test. Vc: gavage 100 mg/kg vitamin C; ITPL‐L: gavage 50 mg/kg insect tea primary leaf; ITPL‐H: gavage 100 mg/kg insect tea primary leaf

Figure 5 shows that the expression levels of Cu/Zn‐SOD, Mn‐SOD, and CAT mRNA in the liver and spleen tissues in the normal group were the highest, whereas expression was weak in the model group. The expression levels of Cu/Zn‐SOD, Mn‐SOD, and CAT in the liver and spleen tissues of mice increased significantly (*p* < .05) after ITPL and vitamin C were administered compared with those in the model group. Moreover, liver and spleen tissue expression was similar between the two groups after ITPL‐H.

### 3.6 Expression of HO‐1, Nrf2, γ‐GCS, and NQO1 mRNA in the liver and spleen tissues

**FIGURE 6:**
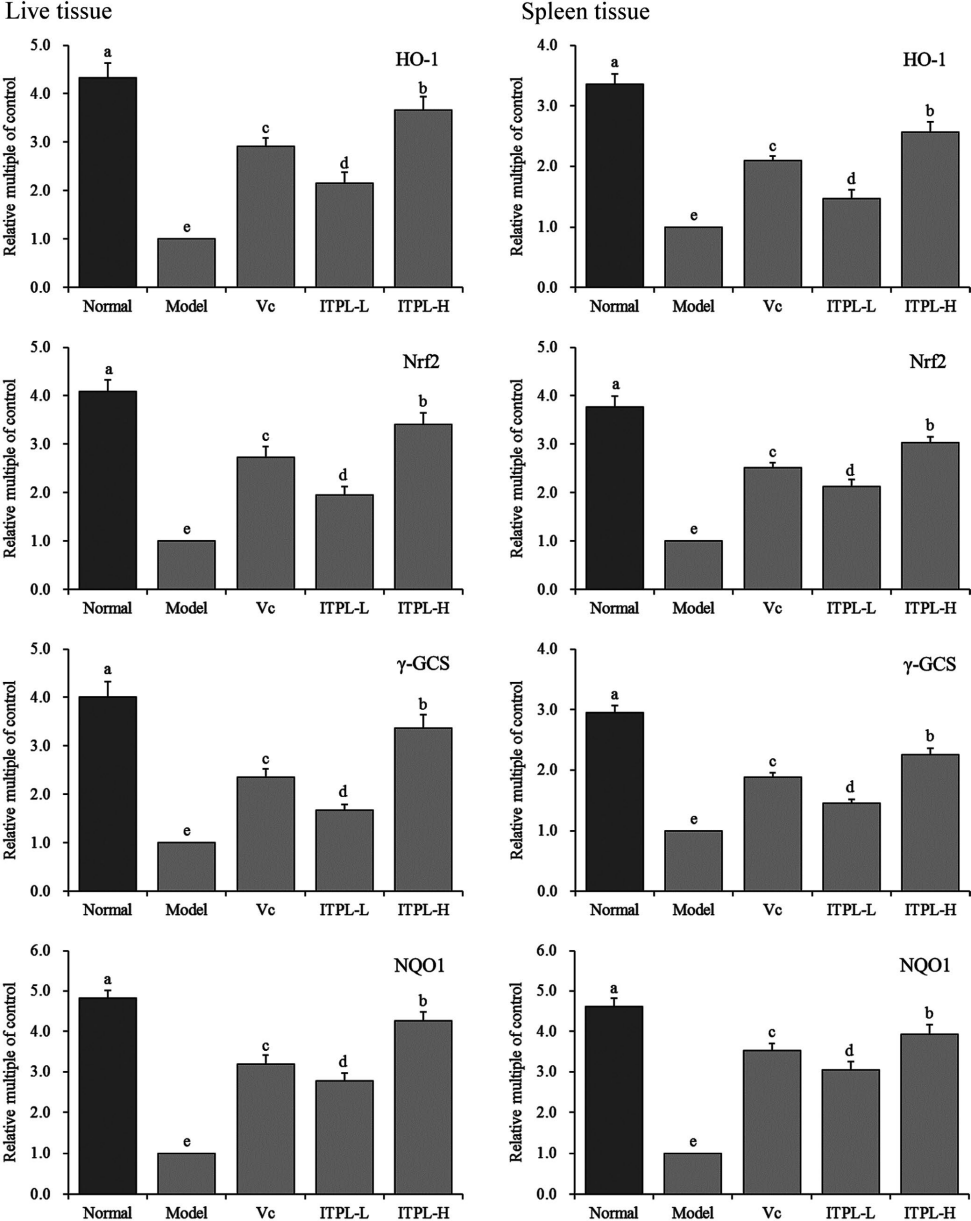
The HO‐1, Nrf2, γ‐GCS, and NQO1 mRNA expression in liver and spleen tissues of mice. ^a–e^Different superscripts indicate significant differences (*p* < .05) according to Duncan's multiple range test. Vc: gavage 100 mg/kg vitamin C; ITPL‐L: gavage 50 mg/kg insect tea primary leaf; ITPL‐H: gavage 100 mg/kg insect tea primary leaf

Figure 6 shows that the mRNA expression levels of HO‐1, Nrf2, γ‐GCS, and NQO1 in the liver and spleen tissues in the normal group were significantly (*p* < .05) higher than those in the other groups, while the expression of HO‐1, Nrf2, γ‐GCS, and NQO1 mRNA levels in the spleen tissues of the model group was the lowest. After D‐galactose was administered, the mice were administered ITPL and vitamin C. The expression levels of HO‐1, Nrf2, γ‐GCS, and NQO1 in the oxidized liver and spleen tissues increased significantly (*p* < .05). The upregulation of these molecules was stronger than those of ITPL‐L and vitamin C.

### 3.7 ITPL ingredients

**FIGURE 7:**
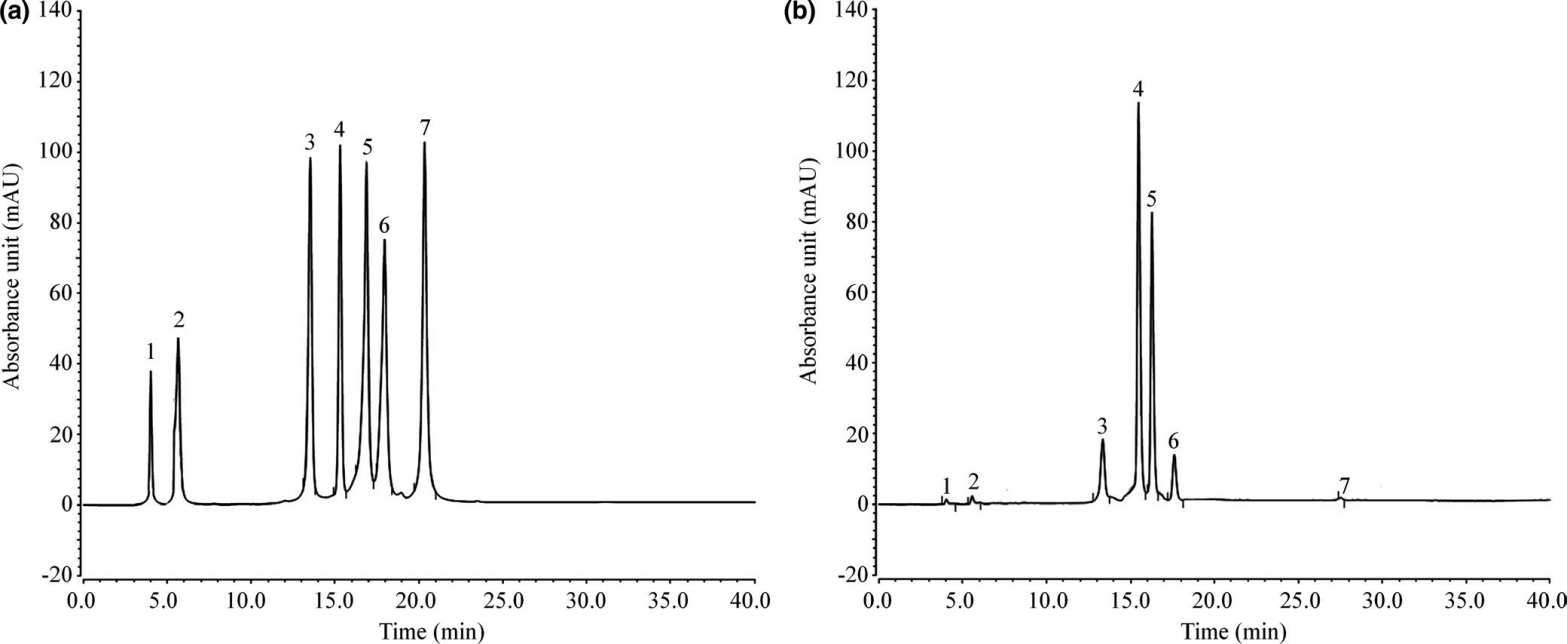
ITPL constituents. (a) Standard chromatograms; (b) ITPLE chromatograms. 1: neochlorogenic acid, 2: cryptochlorogenic acid, 3: rutin, 4: kaempferin, 5: isochlorogenic acid B, 6: isochlorogenic acid A, 7: hesperidin

The experimental results show that ITPL contained the following seven compounds: neochlorogenic acid, cryptochlorogenic acid, rutin, kaempferin, isochlorogenic acid B, isochlorogenic acid A, and hesperidin (Figure 7), with contents of 13.71, 37.44, 116.52, 204.65, 151.17, 50.62, and 11.80 mg/g, respectively.

## 4 DISCUSSION

Changes in the weight and organ indices reflected the status of the body after oxidation in the experimental model. The thymus, brain, liver, kidney, and spleen shrank in size when the body experienced oxidative stress. Such shrinking manifests itself through significant variations in the organ indices. Therefore, the organ indices directly reflect the degree of damage to the organs and serve as a tool to initially evaluate the degree of oxidative stress damage sustained by an animal (Khan, Singer, & Vaughan, 2017; Manini, 2010; Tang & He, 2013). The results of this study showed that D‐galactose caused the murine organ indices to decrease due to oxidation. ITPL alleviated the decrease in the organ indices caused by D‐galactose, thereby mitigating oxidation in the mice.

A large number of excitatory amino acids, such as glutamate, activate the N‐methyl‐D‐aspartate receptor to produce Ca^2+^ influx. When the concentration of Ca^2+^ in cells increases abnormally, nitric oxide synthase is activated to promote the production of NO; NO accelerates Ca^2+^ influx, overloads Ca^2+^ in cells, and directly inhibits the normal physiological activity of mitochondria. Due to the damage caused by the production of $O_{2}^{‐}$ and ·OH, NO reacts with $O_{2}^{‐}$ to form peroxynitrite anions, which decompose into toxic OH^−^ and ${\text{NO}}_{2}^{‐}$ and accelerate cell death (Elíes, Cuíñas, García‐Morales, Orallo, & Campos‐Toimil, 2011). Repeated infections of the body, even if they do not directly involve the central nervous system, result in the synthesis of large amounts of iNOS and overproduction of NO, causing hippocampal neurons to die. Such damage affects memory and accelerates the onset of neurodegenerative diseases, such as Alzheimer's disease. The massive synthesis of iNOS in the anterior pituitary and pineal gland produces NO, which reduce the body's ability to resist stress and reduces the production of melatonin, thereby aging the brain (Khan et al., 2017). Regulating the level of NO has a significant effect on controlling aging. eNOS significantly reduces the number of aortic somatic cells in vivo. eNOS prevents aging of blood vessels caused by oxidative stress, relaxes blood vessels, and protects them (Fukai et al., 2000). Under normal physiological conditions, mechanisms in the nervous system precisely regulate the production, release, diffusion, and inactivation of NO, which is mainly accomplished by regulating the activation and the deactivation of nNOS. In addition to its important functions in the nervous system, nNOS is distributed in skeletal muscle, cardiac muscle, and smooth muscle. In these tissues, NO plays an important role regulating blood flow and muscle contraction (Thomas & Victor, 1998). A decrease in nNOS level generates excessive NO content, which can lead to cerebral ischemic injury and diseases in the elderly (Lee, Hyun, Jenner, & Halliwell, 2001). In this study, ITPL interfered with NO, nNOS, eNOS, and iNOS by maintaining normal levels and avoided the imbalance of NO, nNOS, eNOS, and iNOS levels due to oxidation.

Superoxide dismutase is a metalloproteinase that specifically scavenges superoxide anion radicals ($O_{2}^{‐}$) Wu & Chen (2019). Because they combine with metal ions, the metal ions scavengers can be divided into three types: iron superoxide dismutase, Mn‐SOD, and Cu/Zn‐SOD. They catalyze the disproportionation of oxygen radicals, thus scavenging oxygen radicals and playing an antioxidant role (Bonthius et al., 2015). The superoxide anion radicals produced by the body under normal conditions are necessary for maintaining life activities, but high levels of superoxide anion radicals affect the body by producing oxygen free radicals. Many kinds of physical or chemical stimuli in the external environment produce oxygen free radicals, such as ionizing and ultraviolet radiation. However, SOD eliminates and maintains the balance of oxygen free radicals and prevents bodily damage (Kosenko et al., 2017; Lee et al., 2001). CAT decomposes hydrogen peroxide and is an important antioxidant enzyme in the body, particularly in the liver (Selvaratnam & Robaire, 2016). GSH‐Px is an important peroxidase. The active center of GSH‐PX is selenocysteine, which reflects the level of selenium in the body. Selenium is a component of the GSH‐Px enzyme system. It catalyzes GSH to GSSG and reduces toxic peroxide to nontoxic hydroxyl compounds to protect the structure and active cell membranes from interference and injury by oxidation (Pawlak et al., 1998). GSH is an important regulatory metabolite in cells that has an antioxidant effect and is positively correlated with SOD, CAT, and other antioxidant enzymes in vivo (Berndt & Lillig, 2017; Vázquez‐Medina, Zenteno‐Savín, Forman, Crocker, & Ortiz, 2011). MDA is an end product of lipid oxidation that affects the mitochondrial respiratory chain complex and key enzyme activities in vitro (Patel, Pandya, Clark, Parikh, & Lau‐Cam, 2016). In this study, ITPL significantly regulated oxidized mice by increasing the levels of SOD, CAT, GSH (GSH1 and GSH2), and GSH‐Px, reducing MDA levels, and effectively slowing down the oxidation caused by D‐galactose.

HO‐1 is a prevalent antioxidant enzyme that is usually expressed at a low level. The HO‐1 level increases significantly when the body is stimulated by injury; hence, playing an antioxidant role in tissue repair (Sue et al., 2009). Nrf2, which is located upstream of the antioxidant enzymes, plays a role as a receptor. Under oxidative stress conditions, the Nrf2 level changes prior to other antioxidant enzymes, such as SOD, CAT, and GSH‐Px, which stimulates HO‐1 to play a role (Zhang, Wang, Liu, & Sun, 2015). Nrf2 also directly regulates γ‐GCS. A large number of reactive oxygen species form after the response to imbalanced oxidation/oxidation. Nrf2 becomes activated, resulting in high expression of γ‐GCS, which promotes the synthesis and the activation of GSH and plays an antioxidant role. γ‐GCS is an important rate‐limiting enzyme in the synthesis of GSH. GSH plays a negative feedback role in the synthesis of γ‐GCS. It is impossible to supplement exogenous GSH or its precursor to affect GSH content in cells. The regulation of γ‐GCS is instrumental in the antioxidant role of GSH (Obrador et al., 2014). NQO1, which is downstream of Nrf2, triggers an antioxidant reaction and plays the role of Nrf2 (Jiang et al., 2017). In this study, ITPL upregulated the expression of oxidized Nrf2 by enhancing the expression levels of HO‐1, γ‐GCS, and NQO1, thereby protecting the mice and alleviating oxidative stress.

Neochlorogenic acid, cryptochlorogenic acid, isochlorogenic acid A, and isochlorogenic acid B are chlorogenic acid compounds, which have antioxidant effects when present alone, (Xu, Chen, Liao, Zhang, & Wang, 2015), as they prevent abnormal blood pressure, dyslipidemia, oxidative damage, and nerve injury (Lan, Liu, Chen, & Wang, 2014; Liu, Huang, Niu, Mei, & Zhang, 2019; Sasikala, Rooban, Sahasranamam, & Abraham, 2013; Wang, Wang, Peng, Wang, & Hao, 2016; Wang, Xi, Fan, Cao, & Jiang, 2019; Zhang & Wang, 2007). Rutin is a commonly used antioxidant that scavenges free radicals (Vázquez‐Medina et al., 2011). Anisodamine is an antioxidant that has been detected in a variety of plants with similar antioxidant effects (Chaipech et al., 2012). Quercetin is used as an antioxidant in the pharmaceutical industry (Marunaka et al., 2017). The active substances in this study exerted inhibitory effects on D‐galactose‐induced oxidative damage, which may play a preventive or therapeutic role in certain diseases.

This study showed that ITPL possesses beneficial preventive effects on mice with D‐galactose‐induced oxidation. Their serum, skin, liver, and spleen tissue indices approached normal levels. ITPL demonstrated better results than vitamin C, which is recognized as a powerful antioxidant. ITPL is rich in active substances. Our study results suggest that ITPL could potentially be used as a high‐quality medicine and that it might represent a food source enriched with antioxidants. This study aimed to clarify the mechanism of action of ITPL. However, additional human research is warranted to validate our conclusions.
